# Emotional dysregulation and trauma predicting psychopathy dimensions in female and male juvenile offenders

**DOI:** 10.1186/s13034-016-0130-7

**Published:** 2016-11-01

**Authors:** Kathrin Sevecke, Sebastian Franke, David Kosson, Maya Krischer

**Affiliations:** 1 Clinic for Child and Adolescent Psychiatry, Innsbruck Medical University, Innsbruck, Austria; 2 Faculty II, Developmental Science and Special Education, University of Siegen, Siegen, Germany; 3 Rosalind Franklin University of Medicine and Science, North Chicago, USA; 4 Clinic for Child and Adolescent Psychiatry, University of Cologne, Cologne, Germany

**Keywords:** Psychopathy, Adolescents, Traumatization, Personality pathology

## Abstract

**Background:**

Psychopathy is a specific syndrome that predicts future violent and aggressive behavior in adults. Studies in youth and adults have demonstrated a strong association between early traumatic incidents and later dissocial behavior. Moreover, the impact of personality pathology and emotional dysregulation on aggressive and violent behavior is well established. However, few studies have addressed the relationship between early traumatization and psychopathic traits in adolescents.

**Method:**

The present study examined associations between both general dimensions of personality pathology and early traumatic experiences and the dimensions of psychopathy in 170 male and 171 female adolescent detainees.

**Results:**

Analyses revealed associations between physical abuse, emotional dysregulation and psychopathic traits in delinquent boys but not in delinquent girls.

**Conclusion:**

Hypothesized relationships between trauma, personality pathology could only be confirmed in the lifestyle and antisocial, but not in the core affective and interpersonal facets of psychopathy.

## Background

### Trauma and psychopathy in juveniles

To date, few studies have addressed the relationship between early traumatic experiences and the syndrome of psychopathy in juveniles. However, in addition to adverse family conditions [1], early traumatization is commonly regarded as a causal or mediating risk factor for aggressive and violent behavior [2–4]. As for sex differences, a variety of studies have reported a heightened prevalence of traumatization in female delinquent juveniles compared to males [5–7]. In girls, victimization is often considered an essential risk factor for aggressive behavior [8]. Several researchers have argued that early traumatization has a negative influence on the development of the ability to regulate anger and affect [9, 10] and that it has enduring effects on neural development [11–13].

Several studies have linked child maltreatment with adult psychopathy [14], a syndrome defined by a constellation of features, including affective deficits, interpersonal deceptiveness, and impulsive and antisocial tendencies [15–17]. Lang, Klintenberg, and Alm [18] studied the implications of childhood neglect and/or abuse for adults’ scores on the Psychopathy Checklist (PCL-R) [19] and violent offending. They found that those with more severe victimization histories had higher psychopathy scores than those with less severe victimization histories. Similarly, Bernstein, Stein, and Handelsman [20] reported that, in a substance-abusing sample, physical abuse and physical neglect measured with the Childhood Trauma Questionnaire (CTQ) were related to a latent dimension associated with psychopathic features. Similar results were reported by Weiler and Widom [21]: Victims of childhood abuse and/or neglect had significantly higher PCL-R scores than controls. Furthermore, victimization predicted official and self-reported violence. They suggested that in some individuals the association between early childhood victimization and violence might be mediated by psychopathy. In a Swiss sample of younger male offenders (age 17–27), PCL-R total scores were significantly correlated with the number of prior threatening events experienced [22]. Recently, Verona, Hicks, and Patrick [23] reported that, among female offenders, experiences of both physical and sexual abuse correlated with PCL-R total scores and with scores on the affective-interpersonal and antisocial lifestyle dimensions of psychopathy. However, after controlling for affective factor scores, unique relationships between maltreatment and interpersonal factor scores were no longer significant, suggesting that shared variance between the facets and variance specific to the affective component accounted for the significant zero-order correlations. Marshall and Cooke [14] found in their study comparing childhood experiences of criminal adult psychopaths with non-psychopaths that childhood familial and societal experiences were strongly correlated with PCL-R scores and influenced the adult outcomes.

However, not all studies of adults have reported positive correlations between traumatic environment and psychopathy. In particular, in a sample of 48 male patients in a security hospital in Belgium, Pham [24] found that patients high in psychopathic features reported fewer traumatic events than non-psychopathic patients.

Several recent studies have examined links between psychopathic traits and childhood maltreatment in youth samples, Campbell, Porter & Santor [25] evaluated the clinical, psychosocial and criminal correlates of psychopathic traits in a sample of 226 male and female incarcerated juvenile offenders, using the Psychopathy Checklist: Youth Version (PCL:YV) [26]. They showed that whereas higher PCL:YV scores were associated with having experienced physical abuse, the only psychosocial factor to predict PCL:YV scores was a history of non-parental living arrangements (e.g., foster care). Forth et al. [26] reviewed findings from unpublished doctoral dissertations and reported that several of these studies documented associations between childhood victimization and PCL:YV scores [27, 28]. Also a prior study of Krischer and Sevecke [29] compared a sample of detained adolescents to adolescent students and reported higher indices of traumatization in delinquents. Relationships between physical, but also emotional traumatic experience and the PCL:YV total score could be confirmed among criminal boys, but not among delinquent girls. More recently, Schraft et al. [30] reported correlations between overall childhood maltreatment and psychopathic traits in a sample of adolescent male detainees. In this study, the specific relationship between sexual abuse experiences and psychopathy scores was significant, whereas the relationship between physical abuse experiences and psychopathy scores only approached significance. Kimonis et al. [31] reported that callous-unemotional traits in youth were associated with greater exposure to community violence, and Schraft et al. [30] replicated this relationship, with the latter study demonstrating unique relationships between exposure to community violence and scores on both the interpersonal and antisocial components of psychopathy.

To our knowledge, the only prior study focusing on the relationship between violence, traumatization and psychopathy in delinquent girls was conducted by Odgers, Reppucci, and Moretti [32]. Their results indicated that, although a specific component of psychopathy, deficient affective experience, was related to aggression, the association was no longer significant once victimization experiences were entered into the structural equation modeling (SEM) framework. Odgers and co-workers argued that the psychopathy syndrome in girls is not yet well understood, and their findings raise important questions about the complex relationship between trauma, psychopathy, and aggression in girls. In detail, their findings raise questions about whether many of the important correlates of psychopathic traits in girls are actually consequences of trauma.

### Personality pathology

Personality pathology is another widely recognized factor contributing to offending. Epidemiological studies have identified a high prevalence of personality pathology (40 to 60%) in adult criminal populations in Western societies [33–35]. Moreover, in both women and men, personality disorders are predictive of violent and nonviolent criminal conduct [36, 37]. In adult samples, there are also positive correlations between psychopathy scores and several forms of personality pathology. For example, Hart and Hare [38] reported that, among adult male offenders, psychopathy scores were highly associated with diagnoses of antisocial personality disorder (ASPD) and histrionic personality disorder (HPD). However, they also noted that PCL-R scores correlated positively with prototypically ratings for ASPD, HPD, and narcissistic personality disorder (NPD). Among adult male violent offenders, Huchzermeier et al. [39] found significant relationships between ASPD and borderline personality disorder (BPD) diagnoses and scores on the antisocial lifestyle dimension of psychopathy; they also reported a significant positive correlation between NPD diagnoses and affective-interpersonal scores. Soderstrom et al. [40] showed that, among male offenders, PCL-R total scores as well as affective and lifestyle factor scores were significantly correlated with several Cluster B personality disorder diagnoses. Affective factor scores correlated positively with anxiety and depression and negatively with verbal cognitive ability [41]. Recently, Klipfel, Garafalo and Kosson [42] reported a pattern of unique positive correlations for the interpersonal facet with narcissistic and histrionic PD but no positive correlations with the affective facet and also reported unique relationships for several PDs, including histrionic PD and paranoid PD with the antisocial facet of psychopathy.

Investigators examining personality pathology in adolescents have argued that personality disorders can be reliably and validly assessed in youth 14 years of age and older [43–48]. Although it has been argued that the stability of personality disorders increases from adolescence to adulthood [49] and is lower in adults than previously assumed [50], several longitudinal studies suggest that the stability of maladaptive personality traits relative to age peers may be roughly equivalent in adolescence to that found in adulthood [50–52].

Few studies have examined associations between psychopathic traits and personality pathology in youth samples. However, several authors have reported links between personality disorder symptoms and violence. For example, Johnson et al. [36] reported associations between both Cluster A and Cluster B personality disorder symptoms and violence in a sample of community youth. Lynam and colleagues have demonstrated that callous-unemotional traits in youth are negatively related to scores on the Agreeableness and Conscientiousness dimensions of the Big Five Model of Personality [53, 54]. Moreover, in a sample of 30 adolescent psychiatric inpatients, those who met DSM-III-R criteria for NPD exhibited more psychopathic traits than those who did not meet diagnostic criteria [55]. In addition, patients who met criteria for avoidant or self-defeating personality disorder had lower psychopathy scores than did youth who did not meet diagnostic criteria, while no difference in psychopathy scores was found between those who met criteria for BPD versus those who did not meet criteria for BPD.

The few studies searching for potential sex differences in personality associated with psychopathic traits in adolescence have produced inconsistent findings. Salekin et al. [56] found more dominant and cold trait characteristics in delinquent boys than girls. Focusing on associations of psychopathic traits with delinquency and aggression in a school sample, Marsee, Silverthorn and Frick [57] found no clear sex difference.

On the basis of existing results, however, one cannot decide whether personality traits or personality pathology play a causal role in the pathway to psychopathic features, or whether psychopathy and personality pathology are common outcomes of some other processes, such as parental neglect, social context or genetic predisposition. It is clear that in psychopathic adults, negative emotionality is primarily related to the lifestyle and antisocial dimensions of psychopathy. One recent study indicated that fearlessness and lack of inhibition at age 3 predict higher psychopathy scores in adulthood [58]. Nevertheless, it is premature to state whether temperamental factors and personality traits are related to all dimensions of psychopathy in youth, and it remains possible that observed correlations reflect the influence of other factors not examined.

### Studies of psychopathy dimensions in youth

Factor analyses examining the latent dimensions that underlie the pattern of correlations among PCL:YV item scores have provided acceptable fit for both four-factor [59] and three-factor [60] models in youth samples [61]. Although some studies have reported that these factor models do not fit as well in girls as in boys [60], a recent study has demonstrated that both the three- and four-factor models fit well in a large sample of girls [59]. The four-factor model proposes that psychopathy is best understood in terms of dimensions that reflect interpersonal, affective, lifestyle, and antisocial features. The three-factor model is identical except that it excludes the items comprising the antisocial factor.

Nevertheless, few studies have addressed relations between these dimensions and trauma and personality pathology in youth samples. Moreover, no studies using clinical measures of psychopathy have examined both trauma and personality pathology to characterize psychopathy in female and male detainees.

### The current study

The current study was designed to examine associations between both personality pathology and trauma experiences and the important components of psychopathy. We examined relationships with overall levels of psychopathic traits as well as dimensional ratings for the core and affective and interpersonal dimensions of psychopathy and the less specific lifestyle and antisocial dimensions in incarcerated German adolescents. Well-validated measures of all three constructs were available. We used the Psychopathy Checklist: Youth Version (PCL:YV) [26] to provide reliable estimates of psychopathy traits, including scores on the four facets previously identified in youth samples. We used the Childhood Trauma Questionnaire (CTQ), which measures different forms of trauma: physical abuse, sexual abuse and emotional abuse. To assess personality pathology we used a dimensional measure, the DAPP-BQ (dimensional assessment of personality pathology–basic questionnaire) [62] measuring four higher-order factors of personality pathology (emotional dysregulation, dissocial behavior, inhibitedness, and compulsivity).

To minimize overlap within the domain of personality pathology we focused on the dimension of emotional dysregulation: Kushner et al. [63] recently showed in a hierarchical analysis of the DAPP-BQ that as much as 76% of the variance captured by this instrument is accounted for by an emotional dysregulation factor. They referred to a second dimension that covered the other 24% of the variance as dissocial behavior. However, because the traits that are summarized under dissocial behavior overlap substantially with those encompassed by the psychopathy construct, they provide less useful information about relationships between psychopathy and other forms of personality pathology. Altogether, the dimension of emotional dysregulation combines most traits within the DAPP-BQ and is the factor best reflecting personality pathology in this context.

Judging from the scientific literature, we expected the following:

1. As in prior studies, we expected that emotional dysregulation and sexual and physical trauma experiences would be associated with overall levels of psychopathic traits as well as with scores on several specific components of psychopathic traits in both male and female detainees.
2. We predicted stronger relationships between trauma experiences, emotional dysregulation and psychopathy in delinquent boys than in delinquent girls.
3. We expected different constellations of the relationships between trauma experiences, emotional dysregulation and psychopathy for core versus behavioral dimensions. In particular, we expected the relationship between trauma experiences, emotional dysregulation and psychopathy to be especially strong for the antisocial and lifestyle components of psychopathy.

## Methods

### Participants

This study was conducted as part of the Cologne GAP-Study (Gewalt = violence; Aggression = aggression; Persönlichkeit = personality), an investigation of personality pathology, violence and aggression in adolescents. The sample for this present study consisted of 341 incarcerated juveniles (170 boys and 171 girls), aged 14–19 years (see Table 1). Because in Germany the age of criminal responsibility starts at the age of 14, we selected youth of at least 14 years of age. The boys had a mean age of 17.75 years (SD = 1.16; range = 15–19), the girls a mean age of 17.54 years (SD = 1.33; range = 14–19). The male and female samples did not differ on age (*T* = 1.55; *p* = .122; *d* = .17).

**Table 1:** Characteristics of study population

	Boys	Girls	Total
N (%)	170 (49.9)	171 (50.1)	341 (100)
Age, M (SD)	17.75 (1.16)	17.54 (1.33)	17.64 (1.25)
At least one violent conviction, N (%)	131 (78.4)	102 (60.4)	233 (69.3)
Having lived in foster care, N (%)	115 (67.3)	89 (53.3)	204 (60.4)
*Nationality, N* (%)			
German	124 (72.9)	137 (80.1)	261 (76.5)
Turkish/Arab	9 (5.3)	3 (1.8)	12 (3.5)
Russian–German	5 (2.9)	11 (6.4)	16 (4.7)
African	2 (1.2)	5 (2.9)	7 (2.1)
Others	30 (17.7)	15 (8.8)	45 (13.2)
Violent convictions, *Mdn* (range)	4.00 (1–60)	2.00 (0–14)	3.00 (0–60)
Time of detention [months], *Mdn* (range)	18.00 (2–66)	2.00 (0–42)	9.00 (0–66)

The participants were incarcerated adolescents in two German jails located in the Cologne-Bonn area, North Rhine Westphalia, Germany. During the inclusion phase all incarcerated juveniles were included into the study who met inclusion criteria and who were able to read and understand the German language. In this regard, the investigated sample can be considered representative of the German speaking incarcerated juvenile offenders in this area at this time. Whereas 69.3% of all participants had been sentenced for committing at least one violent criminal act, 30.7% had no violent conviction. Boys and girls differed greatly in this aspect: while 39.6% of the girls had never been convicted of a violent crime, this applies to only 21.6% of the boys (χ_1;95%_^2^ = 12.93; *p* = .001). The mean number of violent convictions for boys was *M* = 5.33 (range = 1–60), for girls it was *M* = 2.47 (range = 0–14). Because the standard deviations are rather high and neither homogenous nor normally distributed, the medians of *Mdn* = 4.00 and *Mdn* = 2.00 (resp.) are more meaningful and were compared using the Mann–Whitney Test (*U* = 7374.0; *n*_1_ = 167, *n*_2_ = 170; *p* < .001). Sample members were on average incarcerated for the second time (SD = 1.84; range = 0–21). The median term of imprisonment for the full sample was 9 months (range = 0–66), whereas boys’ median time of detention was 18.00 months (range = 2–66 months) and girls’ median time of detention was only 2.00 months (range = 0–42 months; Mann–Whitney *U* = 2195; n_1_ = 167, n_2_ = 171; *p* < .001).

The sample consisted of 76.5% Caucasian/Germans, 3.5% Turkish/Arabs, 4.7% Russian–Germans, 2.1% Africans, and 13.2% of other ethnic backgrounds (such as Russian, Polish, Rumanian, Ukrainian) (χ_6;95%_^2^ = 10.74, *p* = .097). The racial/ethnic distribution of participants across sex was equivalent. Comparing the upbringing and family factors, there was no difference in the frequency of divorce or separation of parents among the female (56.1%) versus male (55.1%) adolescents (χ_2;95%_^2^ = 5.16, *p* = .076). However, 67.3% of the boys reported a history of having lived in foster care during upbringing, in comparison to 53.3% of the girls (*χ*_*1;95%*_^*2*^ = 6.8; *p* = .01).

### Procedure

Prior to testing, all participants were oriented to the administration protocol and the nature of the procedure. Under German law parental consent is not required with respect to juvenile matters that involve minimal risk; because all participants were 14 years of age or older, the Legal Administration of Data Protection of the University of Cologne waived parental consent, and the Institutional Review Board of the University Clinic of Cologne gave approval for the current study. Verbal and written explanations of the study were provided to youth prior to testing, and youth were advised that study participation was voluntary. All youth were informed that they could withdraw their informed consent at any time during and after testing. If they had difficulty understanding procedures, additional explanation was provided prior to interviews. The legal status of the participant, the number of convictions and length of incarceration were collected from file information. The protocol excluded juveniles with a schizophrenic spectrum diagnosis, who were under the acute influence of alcohol or other drugs, or who had an IQ lower than 70, determined by a standardized German clinical interview for children and juveniles according to ICD-10 (DISYPS) [64] and by subtests of the Wechsler Adult Intelligence Test [65].

### Measures

*The Psychopathy Checklist Youth Version (PCL:YV)* [26]. The presence of psychopathic traits was assessed with the PCL:YV, a multi-item rating scale that measures interpersonal and affective characteristics as well as overt behaviors. Trained observers rate the presence and severity of each disposition associated with psychopathy based on a semi- structured interview, a review of case history information, and behavioral observation cross-checked with collateral informants. They assigned scores of 0 (absent), 1 (inconsistent), or 2 (present) for each item of the PCL:YV based on the consistency of the evidence for each specific tendency or disposition across different situations. All the scores summed up to a total score (maximum of 40 points). Although a defined cut-off does not exist for the PCL-YV, most authors consider a total score of 25 or 30 or above as pathological with respect to a psychopathic personality. On item basis only a score of two means that the criterion is met. Regarding psychopathy factors, the maximum score for factor 1 and factor 2 is eight and the maximum score for factor 3 and 4 is 10. Researchers suggest that two-thirds of criteria met mean a pathological tendency on factor basis. All authors recommend a dimensional trait analysis of the psychopathy checklist rather than a categorical one [86]. The interview, developed by Forth et al. [26] to assess youth psychopathy, was translated using a forward–backward method and adapted by the authors to the German language, school and legal system [26, 66].

The PCL:YV assessments were carried out by four specially trained professionals with at least master-level education and long-term clinical experience. They received extensive training in administration and scoring of the PCL:YV before conducting the interviews and assessed at least 5 subjects together with one of the two trainers. Afterwards, interrater reliability was re-examined every 10–15 interviews. It was calculated in several ways. First, we compared item scores for all 20 items and reached a kappa score between .52 and .89. Second, PCL:YV total scores were compared, resulting in a kappa score between .80 and .92. The interrater reliability for the males was also compared with that for the females. The interrater reliability for the males/females regarding the single item scores reached a kappa between .66–.90/.55–.79 and for the total score between .84–.93/.76–.88. Reliability studies demonstrated similar levels of interrater agreement (e.g., r = .81 to r = .93) [67–69]. The internal consistency for the PCL:YV item scores was high (α = .89).

*The Childhood Trauma Questionnaire (CTQ)* [20]. Traumatic experiences were measured with the CTQ, a 25-item self-report instrument using a 5-point Likert scale (never, rarely, sometimes, often, and very often true). The questionnaire is designed to inquire about childhood events (“When I was growing up”) in objective, non-evaluative terms. The CTQ contains five scales, labeled Physical Abuse, Emotional Abuse, Sexual Abuse, Physical Neglect, and Emotional Neglect. Five items comprise each of the CTQ´s five maltreatment scales. In addition, three items are used for the Minimization/Denial scale. Whereas emotional abuse refers to verbal assaults on a child’s sense of worth or well-being, physical abuse refers to bodily assaults on a child by an older person, and sexual abuse refers to sexual contact or conduct between a child and an older person. Emotional neglect refers to the failure of caretakers to provide a child’s basic psychological and emotional needs. Physical neglect refers to the failure of caregivers to provide a child’s basic physical needs. CTQ total scores demonstrated good test–retest reliability over a 2- to 6-month interval (intraclass correlation ICC = .88), as well as convergence with the Childhood Trauma Interview. Reliability coefficients ranged from satisfactory to excellent, with the highest for the Sexual Abuse scale (median = .92) and the lowest for the Physical Neglect scale (median = .66). The instrument has been validated for use with adult and adolescent patients.

The German version of the CTQ was translated by the authors, using a forward–backward method. In our juvenile control sample and in the delinquent adolescent sample, respectively, internal consistencies for the five scales were as follows: Emotional Abuse (.83/.85), Physical Abuse (.89/.91), Sexual Abuse (.85/.95), Emotional Neglect (.80/.86) and Physical Neglect (.80/.67). In categorical analyses comparing traumatized versus non-traumatized groups of delinquent juveniles, a CTQ score of 1 (rarely true) or above was used as the cut-off to differentiate the trauma from the non-trauma group, irrespective of the numeric score on the CTQ. This division resulted in a group of non-traumatized individuals (CTQ-score = 0) and a group of traumatized individuals (CTQ-score 1 and above).

*The dimensional assessment of personality pathology-basic questionnaire (DAPP*-*BQ)* [62]. The DAPP-BQ is a 290-item self-report measure with 5 response categories for each item. The items can be summed to yield scores on 18 personality disorder scales. Internal consistency in adult samples ranges from *α* = .83 to .94 (Cronbach’s alpha), and test–retest reliability over a 3-week period ranges from *r*_*tt*_ = .81 to .93 [70]. Principal components analyses yielded four higher-order factors (emotional dysregulation, dissocial behavior, inhibitedness, and compulsivity) underlying the 18 basic traits. This structure was stable across clinical and non-clinical adult samples and was found to be congruent for environmental, genetic, and phenotypic factors based on twin data [70].

For this study, we only used participants’ scores on the first higher-order factor labeled ‘Emotional Dysregulation’ representing unstable and reactive tendencies, dissatisfaction with the self and life experiences, and interpersonal problems. The following traits were consistently found to have their highest loading on this factor: anxiousness, submissiveness, cognitive distortion, identity problems, affective lability, oppositionality, social avoidance, and insecure attachment. For the calculations the mean scores of the higher order factor emotional dysregulation has been computed into the analyses.

The German version of the DAPP-BQ was developed from the original version by the Psychology Department at the University of Bielefeld, Germany, using a forward–backward method and was validated in clinical and non-clinical adult samples [71, 72]. Krischer et al. [48] validated the DAPP-BQ in a non-clinical control juvenile sample and in a delinquent adolescent sample, respectively, and found internal consistencies for the subtraits, allocated to the four higher-order factors: dissocial behavior (.74–.89/.86–.92), emotional dysregulation (.81–.96/.83–.94), inhibitedness (.73/.74–.84) and compulsivity (.86/.87).

### Data analysis

To estimate associations between trauma experiences (abuse), emotional dysregulation and psychopathy, multiple linear regressions were performed using the Generalized Linear Model (GLM) procedure within IBM SPSS Statistics 19.0 (Chicago, IL).

The discrete variables sex, physical abuse and sexual abuse were entered as dichotomous variables; emotional dysregulation, as a quantitative variable. Firstly, the overall psychopathy score served as dependent variable (DV). In a second step for more differentiated and detailed analyses, the four dimensions of psychopathy constituted the outcome variables. All independent variables (IVs) were included in all models irrespective of the strength of their contribution. Two possible interactions were tested for significance; any significant interactions were included in the final model. Assumptions of normal distribution and variance homogeneity within each model were met.

Estimations of effect size are reported using Cohen’s *d* for mean differences; for regression models, the partial *η* ^2^ is computed to provide the proportion of total variability attributable to a factor or interaction, taken as if it was the only variable. We are aware of the problem of partial effect measures [73, 74], but because (1) overall effect size measures such as eta squared or omega squared are less adequate for comparisons across studies [75] and (2) we are interested in the practical significance of separate factors and covariates the calculation of partial *η* ^2^ seems reasonable and most descriptive in our context. However, the positive bias created by this particular measure needs to be considered when interpreting effects [76].

## Results

Table 2 shows the correlations between scores on all variables. Table 3 shows the regression model for overall psychopathy scores as well as for scores on the four sub-dimensions including all independent variables and significant interactions.

**Table 2:** Correlations between all variables

Variables	Physical abuse (6) (N = 166)	Sexual abuse (7) (N = 167)	Emotional dysregualtion (8) (N = 151)
*Females*			
PCL:YV total	.159*	.144^+^	.339***
Interpersonal factor	.003	.124	.069
Affective factor	.035	.063	.097
Lifestyle factor	.121	.094	.372***
Antisocial factor	.240**	.052	.377***
Physical abuse	–	.212**	.250**
Sexual abuse		–	.297***
Emotional dysregulation			–
*Males*			
PCL:YV total	.209*	−.034	.123
Interpersonal factor	.202*	−.064	.169^+^
Affective factor	.113	−.044	.054
Lifestyle factor	.156^+^	−.036	.169^+^
Antisocial factor	.273**	.009	.107
Physical abuse	–	.284***	.315**
Sexual abuse		–	.061
Emotional dysregulation			–

*PCL:YV* psychopathy checklist:youth version total score; *Interpersonal* interpersonal facet score; *Affective* affective facet score; *Lifestyle* lifestyle facet score; *Antisocial* antisocial facet score; *Physical abuse and sexual abuse* subscales on the childhood trauma questionnaire; *EmoDys* emotional dysregulation on the dimensional assessment of personality pathology-basic questionnaire (*DAPP*-*BQ*)

* p < .05, ** p < .01, *** p < .001,^ +^ p < .10

**Table 3:** Regression models for psychopathy and the 4 psychopathy dimensions

DV (corrected *R* ^2^)	IVs	*F*	part *η* ^2^	*ß* (SE)
Psychopathy score (.26)	Sex	71.29***	.24	7.43 (.88)^a^
	Emotional dysregulation	9.73**	.04	1.03 (1.54)
	Physical abuse	5.07*	.02	12.94 (5.75)^b^
	Sexual abuse	.34	.00	.56 (.97)^c^
	Physical abuse × emotional dysregulation	4.51*	.02	4.15 (1.95)
Core dimensions				
Interpersonal (.06)	Sex	17.44***	.07	1.18 (.28)^a^
	Emotional dysregulation	.98	.00	.31 (.31)
	Physical abuse	.32	.00	.16 (.29)^b^
	Sexual abuse	.38	.00	.19 (.31)^c^
Affective (.17)	Sex	49.61***	.18	1.73 (.25)^a^
	Emotional dysregulation	1.40	.01	.32 (.27)
	Physical abuse	.00	.00	.01 (.25)^b^
	Sexual abuse	.01	.00	.02 (.27)^c^
Behavior dimensions				
Lifestyle (.20)	Sex	8.59**	.04	5.14 (1.8)^a^
	Emotional dysregulation	12.89***	.05	.52 (.52)
	Physical abuse	.23	.00	.13 (.27)^b^
	Sexual abuse	.30	.00	.16 (.30)^c^
	Sex × emotional dysregulation	4.08*	.02	1.24 (.61)
Antisocial (.23)	Sex	10.93**	.05	6.61 (2.00)^a^
	Emotional dysregulation	10.04**	.04	.32 (.59)
	Physical abuse	6.99**	.03	.81 (.31)^b^
	Sexual abuse	1.01	.00	.34 (.34)^c^
	Sex × emotional dysregulation	5.62*	.02	1.65 (.70)

*DV* dependent variable; *IVs* independent variables

* *p* < .05; ** *p* < .01; *** *p* < .001

^a^reference category: female

^b^reference category: no physical abuse

^c^reference category: no sexual abuse

In all regression models sex was the strongest predictor of psychopathic traits. Males scored consistently higher on all psychopathy dimensions than females (see Table 4).

**Table 4:** Sex differences for ratings of psychopathy and its dimensions

DV	men, M (SD)	women, M (*SD*)	d
Psychopathy score	25.81 (7.06)	19.86 (6.75)	.86
F1-interpersonal	4.58 (2.24)	3.82 (1.99)	.36
F2-affective	5.05 (1.82)	3.54 (1.85)	.82
F3-lifestyle	6.90 (2.01)	5.71 (2.09)	.58
F4-antisocial	7.46 (1.79)	5.75 (2.63)	.76

In addition, the model addressing overall levels of psychopathy showed main effects for emotional dysregulation and physical abuse as well as their interaction. Interestingly a closer look at this interaction revealed that for individuals who reported no physical abuse the association between emotional dysregulation and psychopathy score proved to be stronger than for individuals reporting physical abuse. The beta-estimate of the interaction term (*ß* = 4.15; SE = 1.95) indicates the difference of the slopes of the regression lines between emotional dysregulation and psychopathy score for the different categories of physical abuse.

With respect to facets, neither scores on the Interpersonal dimension nor on the Affective dimension showed any effects other than sex. Difficulties in emotion regulation were not associated with higher (or lower) scores on these components of psychopathy.

In contrast, the Lifestyle and Antisocial dimensions showed consistent positive associations with emotional dysregulation with effect sizes similar to that of participant sex. Additionally both models revealed significant Sex × emotion dysregulation interactions: In both cases, the relationship between emotional dysregulation and these components of psychopathy were stronger for women than for men, again demonstrated by the size of the beta-estimate of the interaction term (*ß* = 1.24; SE = .61 and *ß* = 1.65, SE = .70, resp.).

Results were similar for indices of traumatic experience. There were no relationships between maltreatment and scores on the interpersonal or affective components of psychopathy. However, there were relationships between traumatic experience and one component of Factor 2. More specifically, only for the Antisocial dimension was a form of maltreatment found to be related to levels of psychopathic traits. In fact, as shown in Tables 2 and 3, the specific relationship between physical abuse and antisocial facet scores was evident at the level of the zero-order correlation as well as in regression analyses that controlled for shared variance with sex and emotion dysregulation scores.

## Discussion

The present study examined emotional dysregulation and trauma as predictors of overall psychopathy scores and scores on the dimensions underlying psychopathy. Unlike most prior studies, we included both female and male detainees to be able to address sex differences within the psychopathy syndrome. With the exception of a few studies with women, research so far has focused on male detainees. Direct comparison between females and males that could be informative about the etiology of psychopathy are rarely examined.

In our study, sex was a strong predictor of the PCL:YV total score and of scores on all four psychopathy dimensions. Consistent with the prior literature [26] incarcerated male adolescents were significantly higher than incarcerated female adolescents on the PCL:YV total score as well as on all four psychopathy dimensions. As illustrated by the beta-estimates the differences in the overall score between persons who report physical abuse versus persons who do not are quite substantial as well. The absolute extent of the coefficient suggests that physical abuse could even have a higher impact in the overall model than sex. This interpretation is put into perspective when considering the beta-estimates within the models explaining the variance in the sub dimensions of psychopathy. In these, the estimates of sex are consistently higher than the ones of physical abuse. Nevertheless, the regression coefficients underscore the importance of the interaction terms in the models and highlight the effects of physical abuse on the one hand and emotional dysregulation on the other hand. In their interpretation, it is important to keep in mind that each coefficient does not explain the total effect on psychopathy of its corresponding variable but that it rather represents the additional effect of adding that variable to the model, if the effects of all other variables in the model are already accounted for.

The differences between boys and girls in the associations between emotional dysregulation and psychopathy contradicted our second hypothesis that relationships between predictors and outcome would be stronger for boys than for girls. More concretely, the interactions between sex and emotional dysregulation suggest that difficulties in regulating emotion may be more strongly related to the Lifestyle and Antisocial dimensions of psychopathy in girls than in boys. In girls, other family-related variables, such as non-parental living arrangements, seemed to be more influential in developing the psychopathy syndrome than traumatization.

Our overall results appear consistent with the first hypothesis that emotional dysregulation and physical traumatization are associated with some components of psychopathy in both male and female detainees. However, a more detailed analysis of the separate regression models reveals that some of these relationships were specific to girls. Moreover, with the exception of the sex differences noted above, the interaction involving overall levels of psychopathic traits was quite distinct from the interaction involving the lifestyle and antisocial components of psychopathy. The fact that sex accounts for most of the variability on the one hand demonstrates the importance of differentiating between male and female individuals when investigating psychopathy. On the other hand, especially when considering the extent of the beta-estimates, our results show that next to the effect of gender, there are still mechanisms that account for specific relations between other variables and psychopathy.

We consider the findings involving overall levels of psychopathic traits first. The overall model shows effects for emotional dysregulation and physical abuse as well as for their interaction. Experiences of physical abuse were associated with higher psychopathy ratings. Similarly, ratings of poorer emotion regulation were associated with higher psychopathy scores. The interaction indicated that the association between emotional dysregulation and psychopathy score held even more for detainees reporting no physical abuse than for participants describing physical abuse during childhood. One can only speculate about the mechanism underlying this particular pattern. The unique effect of physical abuse in the overall model is especially difficult to determine, since it proves significant as a rather strong main effect as well as in the interaction term, where its effect is different at every one of the different values of emotional dysregulation. However, both physical abuse and emotional dysregulation were associated with increased levels of psychopathic traits, but these associations may well reflect largely distinct mechanisms. At the same time, it is important to keep in mind that physical abuse was associated with emotion dysregulation in both male and female youth.

Alternatively, there may be something about the impact of physical abuse that masks the impact of maladaptive emotion regulation or changes its expression. In this study, there was no evidence for any other Abuse X Emotion dysregulation interactions for any component of psychopathy for either boys or girls. Because studies often required larger samples sizes to detect significant interactions, it is possible that the current study was underpowered for assessing the possibility of a three-way interaction involving not only emotion regulation and abuse but also participant sex. However, the samples of males and females in this study were among the largest samples yet employed in studies using clinical measures of psychopathic traits. Moreover, the effect sizes for the sex × abuse × emotion dysregulation interactions were relatively small, it appears unlikely that this interaction reflected a mechanism that was specific to only male or female delinquents.

The relationship between reports of physical abuse and antisocial facet scores is also interesting. Although relationships between physical abuse and the antisocial or lifestyle features of psychopathy (and antisocial personality disorder) have been replicated in various samples in different countries and settings [77, 78], it remains unusual to see a specific correlation with only one of four dimensions underlying psychopathy. Moreover, the proportion of the variance for the antisocial facet was nearly as high as the proportion of variance explained in overall levels of psychopathic traits (corrected *R* ^2^ = .23 vs. .26). We approach this correlation cautiously in recognition that some prior studies have reported similar correlations with environmental factors for the antisocial facet and the interpersonal facet [30]. However, it is remarkable that this association between the antisocial facet and physical abuse was consistent in both male and female adolescent offenders. Moreover, the antisocial facet was the only component to be related to either physical or sexual abuse in youth of either sex. Given that this component of psychopathy reflects early, persistent, and versatile involvement in antisocial activity [79], such findings contribute to a growing literature indicating that this dimension of psychopathy is not simply a measure of conduct problems (or traits related to antisocial behavior) but an index of an important individual differences construct with important developmental implications. Consistent with this perspective, there are now findings linking the antisocial facet of psychopathy to the presence of other erratic dramatic personality disorders and paranoid personality disorder [42, 80].

In line with our third hypothesis, the patterns of relationships were also quite consistent for the two core dimensions of psychopathy as well as for the two behavioral dimensions. First, with respect to the core affective and interpersonal dimensions neither emotional dysregulation nor trauma were meaningful predictors; the corrected *R* ^2^ for the Interpersonal dimension was so small that it seems that individual differences in this dimension likely reflect an entirely separate etiology rather than through effects of emotional dysregulation or traumatization. The corrected *R* ^2^ for the Affective dimension was somewhat higher; still scores on this component of psychopathy were only predicted by the variable sex and not by any of the psychopathological variables tested in this study. This pattern suggests that the core facets are most likely influenced by a set of other variables, such as genetic factors and/or early family factors like early interaction or attachment, which were not addressed in this study.

Results were also similar for the Lifestyle and Antisocial dimensions. Emotional dysregulation has the same importance for explaining these dimensions as sex, and both models reveal a significant interaction of these two variables. In both cases, results indicate a stronger association between emotional dysregulation and variance in these components of psychopathy for girls than for boys. It could well be that girls with more emotional liability show heightened aggressive behavior, stimulation seeking and impulsivity, which are measured by the behavioral factors, but on the other hand are also relatively common among other forms of personality pathology, including borderline personality pathology and antisocial personality disorder without psychopathic features [81, 82]. Hence, these results may be indicative of a gender specific etiology of the traits captured by the behavioral factors, whereas it remains unclear which personality pathology is captured by the behavioral factors. Hicks, vaidyanathan and patrick [83] described a secondary psychopathy subtype (for both men and women) which is similar to an externalizing variant of borderline personality disorder characterized by extreme negative affect and impulsivity; reactive anger, aggression, and violence; substance abuse; trauma; and suicidal behavior. Furthermore, some researchers have argued that secondary psychopathy is one manifestation of a process associated with an impulsive-aggressive behavioral style that is underpinned by weaknesses in neurobiological inhibitory control systems [84, 85].

Contrary to our first hypothesis, sexual abuse was not a significant predictor of psychopathy in this sample. Although there is some evidence from prior studies that sexual abuse may be related to affective deficits of psychopathy [23], but current findings do not appear consistent with these findings. This could be due to the fact, that as reported earlier the prevalence of sexual abuse was comparatively small in our sample. In addition, the CTQ uses a narrow definition of abuse, which only includes exposure to, but not the observation of, abusive acts. Moreover, it is well known that self-reports of experiences of sexual abuse are not always accurate.

## Limitations

This study has several limitations. With respect to measurement, the retrospective nature of reporting trauma experiences presents a number of challenges. Furthermore, the data on trauma experiences and personality pathology were gathered with a self-report measure and were not validated by interview or observational data. In addition, the study was cross-sectional, so all of the relationships reported here are correlational, and any inferences about etiological process are speculative. In any correlational study, it remains possible that an outside factor could account for the relationships reported here. Replication in prospective, longitudinal studies is an important priority for future research.

In addition, the novel findings regarding different mechanisms associated with dysregulation and physical abuse should be regarded as tentative pending replication in an independent sample. In addition, because samples differ not only in the baserates of psychopathic traits but in the extent to which psychopathic traits are correlated with demographic variables, it is important to examine the extent to which relationships that are potentially relevant to the etiology of psychopathic traits generalize across different kinds of samples.

## Conclusion

A high total score on the PCL:YV checklist can be regarded as an indication for outstanding antisocial pathway; however, the total score as a pool for most different antisocial dimensions needs to be interpreted with caution. Both our results and other findings on the heterogeneity of psychopathy suggest that, in some ways, the total score does not seem to be a very useful diagnostic label, whereas the patterns of the core and behavioral facets seem more expedient. Moreover, our results indicate that the heterogeneity of the psychopathy concept is increasingly problematic in an era of developmental psychiatry as we are acquiring increasingly specific treatment methods for specific disorders.

Therefore, based on our findings one would recommend adolescent detainees with high scores on the behavioral dimensions of the PCL:YV and correspondent emotional regulation deficits to attend an anti-aggression-training in order to improve impulse control deficits and to handle traumatic experiences. In adolescents with high scores on the psychopathy dimensions, these therapeutic objectives seem to be displaced. Instead, it should be focused on the pathological personality dimensions while new therapeutic strategies for these specific characteristics ought to be developed.

Current results and as well as other recent findings pointing to distinct subtypes of individuals with psychopathic traits and distinct correlates for some of the different components of psychopathy appear consistent with recent arguments that the psychopathy total score may be less informative than the profile of scores on the core and behavioral facets. On the other hand is the heterogeneity of the psychopathy concept increasingly problematic in an era of developmental psychiatry where we are acquiring increasingly specific treatment methods for specific disorders.

## Abbreviations

ASPD: antisocial personality disorder
BPD: borderline personality disorder
CTQ: childhood trauma questionnaire
DAPP-BQ: dimensional assessment of personality pathology
HPD: histrionic personality disorder
NPD: narcissistic personality disorder
PCL-R: psychopathy checklist
PCL: YV psychopathy checklist: youth version
PD: personality disorder
SEM: structural equation modeling
